# Intra-Arterial Melphalan Chemotherapy for Retinoblastoma in a Developing Nation: Real-World Outcomes and Prognostic Factors

**DOI:** 10.3390/cancers17121955

**Published:** 2025-06-12

**Authors:** Yacoub A. Yousef, Mona Mohammad, Odai Al-Jabari, Farah Halawa, Lama Al-Fahoum, Hadeel Halalsheh, Jakub Khzouz, Maysa Al-Hussaini, Imad Jaradat, Mustafa Mehyar, Robert Rejdak, Mario Damiano Toro, Hazem Haboob, Ibrahim Al-Nawaiseh

**Affiliations:** 1 Department of Surgery (Ophthalmology), King Hussein Cancer Centre (KHCC), Amman 11941, Jordanfarah97209@gmail.com (F.H.); lama.anmar@hotmail.com (L.A.-F.); mustafamehyar@hotmail.com (M.M.); i-nawaiseh@hotmail.com (I.A.-N.); 2 Department of Interventional Radiology, King Hussein Cancer Centre (KHCC), Amman 11941, Jordan; oa.14728@khcc.jo (O.A.-J.); dr_hazem@hotmail.com (H.H.); 3 Department of Pediatrics Oncology, King Hussein Cancer Centre (KHCC), Amman 11941, Jordan; hadeelhalalsheh@khcc.jo; 4 Department of Pathology, King Hussein Cancer Centre (KHCC), Amman 11941, Jordan; jkhzouz@khcc.jo (J.K.); mhussaini@khcc.jo (M.A.-H.); 5 Department of Cell Therapy and Applied Genomics, King Hussein Cancer Centre (KHCC), Amman 11941, Jordan; 6 Department of Radiation Oncology, King Hussein Cancer Centre (KHCC), Amman 11941, Jordan; ijaradat@khcc.jo; 7 Chair and Department of General and Pediatric Ophthalmology, Medical University of Lublin, 20-059 Lublin, Poland; robertrejdak@yahoo.com; 8 Eye Clinic, Public Health Department, University of Naples Federico II, 80138 Naples, Italy

**Keywords:** intra-arterial chemotherapy, globe salvage, melphalan, retinoblastoma

## Abstract

**Background:** Intra-arterial chemotherapy (IAC) is increasingly useful for treating intraocular retinoblastoma (Rb). It offers targeted delivery of chemotherapy with reduced systemic exposure. In this study, we evaluate management outcomes and identify predictive factors for globe salvage following IAC in children with Rb. **Methods:** This retrospective study included 20 eyes of 20 melphalan-based IAC-treated patients (67 sessions) between 2015 and 2023 in a tertiary cancer center (King Hussein Cancer Center) in Jordan. Data collection included patients’ demographics, tumor staging, eye salvage, complications, and survival, followed by statistical comparisons between eye salvage rates and clinical factors. **Results:** The median age of IAC initiation was 38 months (range: 6–78 months). IAC was used as a primary treatment in 35% (7/20) of eyes and as a secondary treatment following systemic chemotherapy in 65% (13/20) of eyes. Nineteen (95%) eyes showed initial tumor regression, 15 (75%) eyes showed short term tumor control, and long-term eye salvage was achieved in 11 (55%) eyes. Poor prognostic factors for eye salvage included advanced tumor stage (Group D/E: 43% salvage rate vs. Group C: 83%; *p* = 0.047), vitreous seeding at the time of IAC (38% with seeding vs. 75% without; *p* = 0.046), use of IAC as a secondary rather than a primary treatment (46% vs. 71%; *p* = 0.047), and the need for >3 IAC cycles (20% success with >3 cycles vs. 67% with ≤3 cycles; *p* = 0.034). Complications were notable: systemic adverse effects were seen in five (25%) patients, including neutropenia (20%) and bronchospasm (6%). Procedure-related complications were seen with 22% of injections, including failure of the procedure (7%), ophthalmic artery spasm (6%), and intra-procedural stroke (3%). Five (25%) eyes developed ocular complications, including vitreous hemorrhage (15%), retinal detachment (10%), optic atrophy (10%), and retinal or choroidal ischemia (10%). Notably, all infants under 12 months of age (4/4) developed complications, including the two events of stroke. At a median follow-up of 60 months, eye salvage was achieved in 11 (55%) eyes, and none of the 9 (45%) enucleated eyes showed high-risk pathological features. There was no orbital recurrence, and one (5%) child developed CNS metastasis and passed away. **Conclusion:** IAC achieves long-term globe salvage in 55% of Rb cases; however, outcomes are poorer with Group D/E tumors, vitreous seeds, prior IVC failure, or requiring >3 IAC cycles. While reducing systemic chemotherapy toxicity, IAC carries significant risks of vision- and life-threatening complications. Infants and single-eyed patients require particularly cautious consideration. Though IAC remains crucial for globe preservation, optimal implementation demands improved patient selection criteria, multicenter collaboration, and long-term outcome studies to maximize safety and efficacy.

## Simple Summary

Intra-arterial chemotherapy (IAC) has emerged as a targeted treatment for intraocular retinoblastoma, minimizing systemic toxicity. This retrospective study evaluated 20 eyes treated with melphalan-based IAC (67 sessions) at King Hussein Cancer Center (2015–2023). The median age at treatment was 38 months, with IAC used as primary therapy in 35% of eyes and secondary therapy (post-systemic chemotherapy) in 65%. Initial tumor regression occurred in 95% of eyes, but long-term globe salvage was achieved in only 55%. Poor prognostic factors included advanced tumor stage (Group D/E: 43% salvage vs. Group C: 83%), vitreous seeding (38% vs. 75% without seeds), secondary IAC use (46% vs. 71% primary), and requiring >3 IAC cycles (20% vs. 67% success with ≤3 cycles). Complications were significant: 25% of patients experienced systemic adverse effects (neutropenia, bronchospasm), while 22% of injections led to procedural issues (artery spasm, stroke). Ocular complications (25% of eyes) included vitreous hemorrhage, retinal detachment, and ischemia. Notably, all infants under 12 months developed complications, including two strokes. At 60-month median follow-up, no enucleated eyes had high-risk pathology, and one child died from CNS metastasis. While IAC preserves globes in 55% of cases, its risks—especially in advanced disease, infants, or salvage settings—highlight the need for stricter patient selection, multicenter collaboration, and long-term safety data to optimize outcomes.

## 1. Introduction

Retinoblastoma (Rb) is the most common primary intraocular malignancy in children, with an incidence of one in 15–20 thousand live births [1,2]. Recent advances in the treatment of Rb have significantly improved globe salvage rates. This is due to the use of multiple treatment modalities for Rb in order to avoid enucleation, including systemic chemotherapy (IVC), intra-arterial chemotherapy (IAC), intravitreal chemotherapy (IViC), cryotherapy, laser therapy, and plaque radiotherapy; however, each treatment modality has specific benefits and risks [3,4,5,6,7,8].

IVC is used most widely as the primary treatment for Rb, in combination with laser photocoagulation, cryotherapy, and thermotherapy [3,4,5,6,7,8]. The initial success of IAC, administered via a microballoon and guiding catheter, was first reported by Suzuki and Kaneko [9]. This was later popularized by Abramson et al. [10], where IAC was administered using the superselective ophthalmic artery infusion technique (chemosurgery). Thereafter, over the past decade, IAC became a popular treatment modality for Rb and became the primary treatment in some of the developed countries [10,11]; its use is still limited in the developing countries due to limitations in infrastructure and expertise as well as the high cost [12,13]. By delivering higher drug concentrations directly to the tumor, IAC has been used as a rescue therapy in cases of recurrent or persistent Rb after initial treatment with IVC, with success rates ranging from 57% to 67% [14,15,16]. IAC has shown improved outcomes, particularly in patients with Group D and E Rb [17].

On the other hand, many centers still use IAC with caution because of the associated systemic and ocular complications as well as the possible but not evident increased risk of metastasis [18]. The most frequent ocular complications of IAC are retinal and/or choroidal vascular, ischemic, or atrophic effect, documented in 5–17% of cases that may risk the vision in eyes with normal macula mainly in single-eyed patients [18,19,20]. Herein, we are evaluating the outcome and the factors affecting the outcome of using IAC as a primary or secondary treatment for intraocular Rb in a developing country.

## 2. Methods

This is a retrospective study of 20 eyes from 20 patients who had a clinical diagnosis of intraocular Rb and were treated with IAC (melphalan). The study period spans from January 2015 to December 2023. The Institutional Review Board at KHCC approved the study (25KHCC001).

Each patient underwent a comprehensive ophthalmic examination performed under general anesthesia that included fundus photos taken by the RetCam system (Natus Medical Incorporated, Pleasanton, CA, USA) for documentation. Selection required access to patients’ medical, radiological, and pathological reports and fundus RetCam images. Data collected included each patient’s age, gender, family history, laterality, age at diagnosis, disease stage, treatment modalities, follow-up, eye salvage, complications, and survival.

**Inclusion** **and exclusion criteria**

Only the eyes with the clinical diagnosis of Rb that were treated at one stage by IAC and were followed for at least 1 year after the last IAC procedure were included. Eyes that were followed for less than one year were excluded. The IAC procedure was offered as a primary or secondary treatment to patients who were older than 6 months of age with intraocular Rb with an absence of anterior segment invasion and with an absence of extra-scleral or optic nerve invasion.

**Clinical Characteristics and Definitions**

Tumors were staged at presentation according to the IIRC and 8th edition TNM staging systems [21,22]. IAC was used as a primary treatment for intraocular Rb or as a secondary treatment after failure of control by systemic chemotherapy combined with focal consolidation therapy (including trans-pupil thermal therapy, cryotherapy, and radioactive plaque therapy). Failure of treatment after IAC was defined as any uncontrollable tumor progression or relapse after IAC that ended with a team decision of enucleation. Enucleation following IAC was indicated in cases of: (1) persistent tumor progression despite IAC; (2) extensive vitreous seeding where IViC was not safely feasible; and (3) persistent massive intraocular hemorrhage precluding adequate tumor assessment. Visual acuity for children was evaluated using an LEA chart when possible.

**Previous treatment**

The patients who received IAC as a secondary treatment had received a combination regimen of chemotherapy that consisted of CVE (carboplatin, vincristine, and etoposide). Each CVE cycle was repeated every 3–4 weeks for a total of 6 to 8 cycles according to the patient’s condition and tumor status. Ocular oncology follow-up was provided as examination under general anesthesia before each cycle of chemotherapy and every 4 to 8 weeks thereafter. Fundus photos were taken using a RetCam II (Clarity Medical System, Pleasanton, CA, USA), and focal therapy was provided using thermotherapy and/or cryotherapy.

**Protocol for Intra-Arterial Melphalan**

Melphalan hydrochloride (alkylating agent) is commercially available as 50 mg of a lyophilized powder, reconstituted with a preservative-free sterile 0.9% sodium chloride solution. Selective intra-arterial melphalan injection is administered by interventional neuroradiology every 4 weeks for 3 injections. Doses are weight dependent at 0.35–0.42 mg/kg in 30 mL over 30 min to a maximum of 3 mg (patients 6–12 months old), 4 mg (patients 1–3 years old), or 5 mg (patients > 3 years old).

Every treated eye is assessed 3–7 days before the next injection by examination under anesthesia. Eyes that show no response after the third injection are not treated any further with AIC. Eyes with complete response will not receive any further IAC injections, but those with partial response will be considered by the team for further treatment of up to 8 IAC injections. Treatment is suspended in the case of massive vitreous hemorrhage or life-threatening complications such as stroke.

**Interventional Radiology Technique for IAC**

All IAC procedures were performed under general anesthesia by experienced interventional neuroradiologists using femoral artery access. A 4F or 5F sheath was placed in the femoral artery, and a guiding catheter was navigated into the internal carotid artery under fluoroscopic control. Superselective catheterization of the ophthalmic artery was then performed using a microcatheter (1.2–1.5F) with roadmapping and real-time digital subtraction angiography (DSA) guidance. Once optimal positioning was confirmed angiographically, melphalan was infused manually in a pulsatile manner over approximately 30 min diluted in 30 mL of saline. Meticulous care was taken to ensure the catheter tip was appropriately positioned at the ostium of the ophthalmic artery to avoid reflux or unintended perfusion of anastomotic branches.

Technical challenges encountered included ophthalmic artery spasm, catheterization failure, and inadvertent entry into anastomotic vessels such as the middle meningeal or infraorbital artery. In these cases, repeat angiographic evaluation was used to reposition the catheter or determine if alternate routes were feasible. Procedures were aborted if secure, targeted delivery into the ophthalmic artery could not be achieved. Vasospasm was managed conservatively by pausing the procedure and reattempting with vasodilators when necessary. Careful angiographic mapping and real-time monitoring throughout the procedure were essential to ensure effective drug delivery and minimize off-target complications.

**Statistical Analysis**

Statistical analysis of tumor control and eye salvage was correlated to demographics and tumor features. The *p* value was measured to test the predictive power of each factor using the exact Fisher test, and a *p* value of less than 0.05 was considered significant.

## 3. Results

### 3.1. Demographic and Clinical Features

This study included 20 eyes from 20 patients diagnosed with intraocular Rb and who received a total of 67 IAC melphalan injections. The mean age at diagnosis was 21 months (median 19 months; range 1–72 months), and the mean age at the time of IAC was 40 months (median 38 months; range 6–78 months). Of all the patients, 7 (35%) were male and 13 (65%) were female. Nine patients (45%) had bilateral disease, and one (5%) had a positive family history of Rb. All patients received intra-arterial melphalan injections, and none received bilateral IAC in this series (Table 1).

### 3.2. Tumor Features and Number of Injections

Based on the International Intraocular Retinoblastoma Classification (IIRC), there were 6 eyes (30%) in Group C, 13 eyes (65%) in Group D, and 1 eye (5%) in Group E. As per the 8th edition TNM staging system, the majority of eyes (95%) were Group T2: 2 eyes (10%) T2a, 17 eyes (85%) T2b, and 1 eye (5%) T3 (Table 1). The eye in Group E (T3) belonged to a patient who had previously undergone enucleation of the other eye. The patient had a large tumor that occupied more than half of the eye and had neovascular glaucoma at diagnosis and treatment. The indication for IAC was primary in 6 (30%) eyes (all of which had unilateral Group D disease) and secondary in 14 (70%) eyes. The number of injections differed between patients, with most of them (75%) receiving 3 cycles or less (Table 1).

### 3.3. Treatment Outcomes and Eye Salvage

Twenty eyes underwent 67 IAC procedures. Five procedures failed (three had severe ophthalmic artery spasms, one had a severe bronchospasm, and one had a stroke before the injection), while 62 injections successfully delivered chemotherapy to the eye. Eleven (55%) eyes received three injections; three (15%) eyes received four injections; and two (10%) eyes received five injections. Four (20%) eyes could not complete three injection protocols; one (5%) eye received only one injection, after which, it developed a massive vitreous hemorrhage that resulted in enucleation; and three (15%) eyes received only two injections (two patients had a stroke and the team decision was not to give any more injections; one patient had a bronchospasm, so the family refused to allow a third injection. Of interest, two of these three eyes had tumor control).

**Figure 1:**
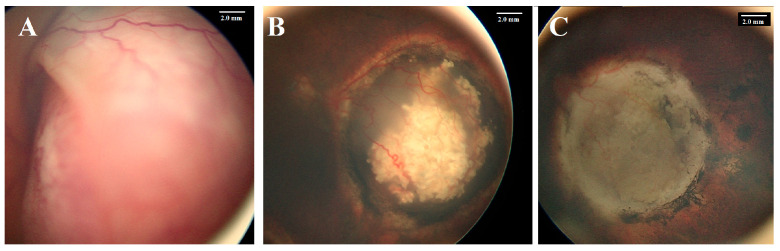
A 1-year-old girl presented with bilateral retinoblastoma. The left eye had a Group D tumor (**A**) with total exudative retinal detachment. Following six cycles of systemic chemotherapy combined with focal therapy, the tumor showed partial regression (**B**). Intra-arterial chemotherapy (IAC) induced further regression, and the tumor was totally inactive after three IAC cycles (**C**). This response was maintained over 6 years of follow-up.

**Figure 2:**
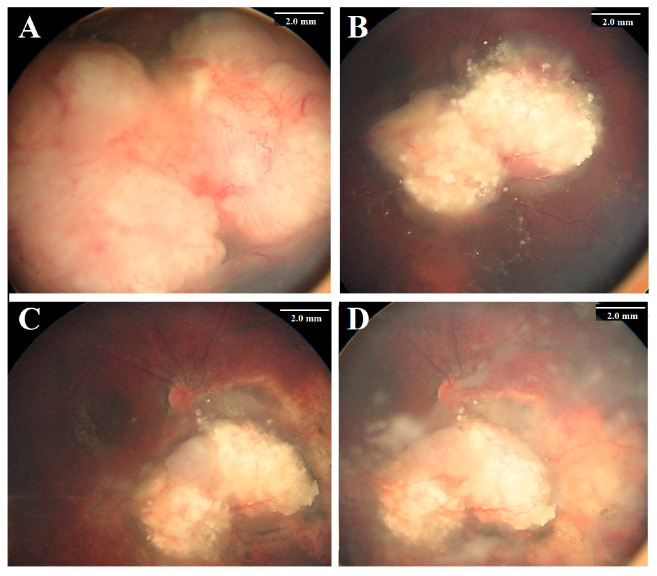
A 2-year-old girl presented with bilateral retinoblastoma. The right eye had a Group D tumor (**A**) with extensive vitreous seeding. Following six cycles of systemic chemotherapy, the tumor showed partial regression (**B**). Intra-arterial chemotherapy (IAC) induced further regression after the first two cycles (**C**), but after the third cycle, the tumor recurred aggressively, with prominent vitreous seeding (**D**). Despite two additional IAC cycles, no further improvement was observed. Due to the lack of a safe quadrant for intravitreal chemotherapy, the eye was ultimately enucleated.

All but one eye (which developed a massive hemorrhage after the first injection) (95%) showed tumor regression following the first injection. Tumor control by IAC was achieved in 15 (75%) eyes; however, with long-term follow-up, eye salvage was achieved in 11 (55%) eyes, while 9 (45%) eyes were enucleated because of persistent tumor activity or tumor recurrence. None of the enucleated eyes showed high-risk pathological results (HRPF) (Figure 1 and Figure 2).

At the date of the last available follow-up (mean, median: 62, 60 months; range: 14 to 100 months), there were no orbital recurrences. However, one patient, who had had the other eye enucleated overseas before referral (with no available pathology report), had the tumor in her single eye treated successfully with IAC. Unfortunately, this patient developed CNS metastasis and passed away (Table 2).

The significant poor predictive factors for eye salvage by IAC were Group D or E at diagnosis, concomitant active vitreous seeds at the time of IAC, secondary treatment after failure of systemic chemotherapy, and the need for more than 3 IAC injections to control the tumors (Table 1). Age at the time of IAC, gender, family history, laterality, TNM stage, and presence of subretinal seeds were not considered predictive factors for tumor control (Table 1). Among 8 eyes presenting with vitreous seeds at the time of intra-arterial chemotherapy (IAC), 5 did not receive intravitreal chemotherapy (IViC) due to either unavailability of the treatment modality (n = 2) or absence of a safe injection site (n = 3). Of the 3 eyes that underwent IViC, 2 eyes achieved disease control while 1 eye failed treatment. Notably, 1 eye without IViC administration demonstrated successful tumor control with IAC alone.

### 3.4. Complications

**Figure 3:**
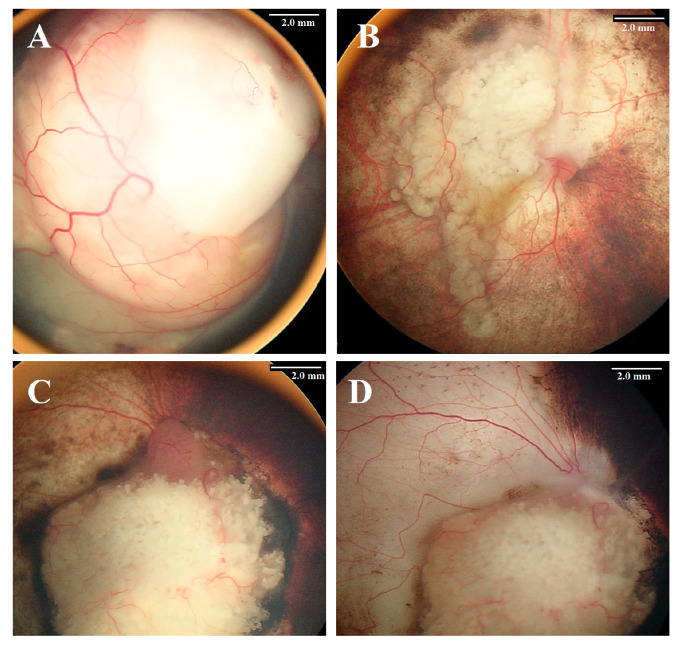
The first patient (**A**) underwent primary intra-arterial chemotherapy (IAC), which resulted in severe chorioretinal ischemia (**B**). Similarly, the second patient (**C**) received IAC following tumor recurrence after systemic chemotherapy. While the tumor was successfully controlled (**D**), the retina developed significant ischemic damage.

IAC complications were categorized as systemic, procedure related, and ocular (Table 2). Systemic complications occurred in five patients (25%), with neutropenia observed in four (20%) and skin erythema in one (5%). Procedure-related complications were reported in 15 of 67 procedures (22%), including failure of the procedure in 5 cases (7%), ophthalmic arterial spasm in 4 cases (6%), strokes in 2 cases (3%), and bronchospasm in 4 cases (6%), with 1 procedure being aborted due to this complication. Ocular complications (Figure 3) were noted in 5 eyes (25%), with subretinal hemorrhage in 2 eyes (10%) and tumor hemorrhage in 3 eyes (15%), one of which led to enucleation due to persistent hemorrhage after the first IAC injection. Additionally, optic atrophy and retinal detachment were each observed in 2 eyes (10%), severe choroidal and retinal ischemia in 2 eyes (10%), peripheral chorioretinal atrophy in 3 eyes (15%), and oculomotor palsy in 1 eye (5%). Of interest, all four patients who were younger than 12 months at the time of IAC showed at least one complication, including 2 (50%) strokes, 2 (50%) ophthalmic artery spasms, and 3 (75%) instances of some kind of ocular ischemia or hemorrhage.

## 4. Discussion

Retinoblastoma management requires a multimodal approach. While systemic chemotherapy combined with focal consolidation remains the first-line treatment at most centers worldwide, IAC is a promising treatment that emerged as both: (1) an effective salvage therapy for chemoresistant cases and (2) a primary treatment option for select intraocular Rb cases. The direct delivery of chemotherapy into the ophthalmic artery in IAC allows for higher local drug levels with reduced systemic exposure. In this report, we present our institution’s experience with 20 eyes treated with melphalan-based IAC, comprising a total of 67 injections. Our overall eye salvage rate was 55%, which was at the lower end of the range cited in the literature (58% to 80%) [12,13,14,15,16,23].

Eye salvage rates vary in the literature by disease severity but are consistently favorable; however, the literature is full of mixed reports where there is an overlap between tumor response rate and ultimate eye salvage rates. Some reports showed high initial response rates with no clear conclusions about the long-term eye salvage rate. Muen et al. reported 80% tumor control with single-agent melphalan IAC in previously treated eyes [23], and Li et al. [24] reported 78% eye salvage following salvage IAC. In a large retrospective study from Brazil involving 357 eyes treated over 13 years, the overall eye salvage rate reached approximately 85%, with a 70% salvage rate in Group D eyes and 40% in Group E eyes [19]. Shields et al. reported globe salvage in 81% of Group C, 78% of Group D, and 35% of Group E eyes following IAC [25]. Abramson et al. reported a 67% eye salvage rate in Group D eyes [26,27], and Kaliki et al. reported salvage in 58% of bilateral advanced (Group D or E) eyes in a cohort from India [12]. Francis et al. [28] described 83% 24-month ocular survival in eyes receiving a second IAC course [24,28]. These results and the overall discrepancy in outcomes underscore the reality that while IAC can be very successful, its success is contingent upon multiple interrelated clinical factors. This discrepancy may be due to differences in the stage of the tumor at diagnosis and if this patient failed previous therapy or was treated primarily by IAC.

Our comparatively modest eye salvage rate is likely due to the high proportion of advanced disease in our population: 75% of eyes in our series were Group D or E at diagnosis. Eyes in Groups D and E have a known poor prognosis due to massive tumor burden and the presence of resistant vitreous and/or subretinal seeds [23,24,25,28,29,30,31,32]. Furthermore, 65% of our patients were treated with IAC as a salvage therapy following the failure of intravenous chemotherapy (IVC), a situation that is known to be associated with worse outcomes. These findings are in agreement with earlier reports that secondary IAC, particularly in eyes with massive vitreous seeding, are less likely to result in persistent long-term tumor control [24,31]. Our study showed that both prior systemic chemotherapy and seeds in the vitreous were statistically significant poor predictors of eye salvage (*p* = 0.047 and 0.046, respectively). Furthermore, the need for more than three IAC cycles to control the tumor was shown to be a poor prognostic factor as well. Eyes that mandated more than three injections to be controlled had a much poorer salvage rate (20%) compared with those controlled with three or fewer (67%). This suggests that most of the tumor response should be detected during the first three cycles, following which the residual tumor cells are expected to be resistant or have a more aggressive biology.

Complications in our study were not trivial. While systemic toxicity was relatively unusual, it was limited mostly to mild neutropenia (20%) and skin erythema (5%). Side effects related to the procedure were encountered in 22% of injections, including spasms of the ophthalmic artery (6%), bronchospasm (6%), and stroke (3%). Five procedures also failed due to technical or systemic complications. Of interest, all of the infants younger than 12 months of age had at least one complication, and two of them experienced strokes, highlighting the higher risk of the procedure in very young children with a smaller vessel size and a more vulnerable physiology. Ocular complications were frequent and clinically significant as well. We observed subretinal hemorrhage (10%), tumor hemorrhage (15%), retinal detachment (10%), and optic atrophy (10%). In one case, a massive hemorrhage after the first injection led to enucleation because of failure to evaluate the tumor activity after this treatment. A few cases also demonstrated signs of retinal or choroidal ischemia causing loss of vision even in anatomically intact eyes. These complications have also been reported by earlier studies. Muen et al. reported several local side effects which included third cranial nerve palsy (40%), orbital edema (20%), permanent retinal detachment (7%), and vitreous hemorrhage (27%); in 47% of the cases, retinal pigment epithelium changes developed [23]. Furthermore, the literature review exhibited secondary cancers, or lasting neurological sequelae associated with IAC [25,33], as we encountered 2 cases of stroke.

The most common adverse effects reported were transient, including fever, nausea, and periprocedural hemodynamic instability, which were promptly resolved with the proper management [33,34]. Local eye complications, such as eyelid edema and ptosis, tended to improve within weeks to months [35]. However, more serious complications such as choroidal ischemia were also observed, and, because this can result in permanent vision loss, it was of particular concern for eyes with good visual potential [3,36]. Moreover, stroke is a life-threatening complication, and even though our 2 cases with stroke passed without consequences, it is still a very dangerous life-threatening side effect.

Complications appear to be related to both technical factors as well as drug toxicity. Studies have shown that more subtle techniques, including the use of smaller catheters and more speedy drug administration, can reduce risks [34]. The position of the catheter in the ophthalmic artery was found to be a significant factor in complications like choroidal ischemia [36]. While others consider drug dosage (particularly age-adjusted melphalan) to also be a significant factor [33], it is the level of experience with performing subtle techniques that appears to be even more significant, with centers reporting fewer complications as their technique becomes more refined. Dalvin et al. showed that the vascular events decreased from 59% to 9% with increasing experience [37]. Radiation exposure from fluoroscopy remains a concern, particularly in children who carry Rb1 gene mutations, but optimized protocols have demonstrated significant dose reduction [38,39,40]. Of interest is that none of the eyes enucleated in our series contained high-risk histopathological features and no orbital recurrences have been reported.

While IAC has improved eye salvage in Rb, it lacks systemic chemoprotective effects, and concern for occult micrometastasis persists, particularly in Group E eyes with high-risk features [18]. Eye salvage is only significant if achieved without compromising survival. Yousef et al. reported 2.1% metastasis after IAC in a systematic review, most of which were Group E eyes with high-risk features [18]. Manjandavida et al. also cautioned that systemic chemotherapy is still required in these instances to reduce metastatic risk. Careful patient selection, particularly not attempting IAC monotherapy in eyes with poor visual prognosis or signs of high risk, is the key to both life and vision preservation [41].

The limitations of the current study include its small number of patients and relatively short follow-up duration. Late recurrence or metastasis as late results cannot be characterized in detail. Future multicenter studies with larger patient groups and longer observation durations should provide better-defined optimal indications and methods for IAC.

## 5. Conclusions

This study supports the existing evidence base for IAC as a valuable tool in Rb management. Melphalan IAC has expanded the therapeutic options for intraocular Rb, particularly in refractory or advanced disease. Successful tumor control in a subset of eyes was obtained by IAC in our series, with an eye salvage rate of 55%. Poor prognosis factors were Group D/E disease, vitreous seeding, secondary use after IVC failure, and a need for more than three IAC injections. While IAC spares systemic toxicity, it does not possess the systemic chemoprotective activity of intravenous chemotherapy, which creates concern regarding occult micrometastasis, especially in eyes with high-risk characteristics. Metastasis, although infrequent, continues to represent a life-threatening event and highlights the necessity of extensive patient examination and systemic follow-up. Our findings confirm the need for caution in infants and in cases of unfavorable initial response to IAC. With advanced skill and ideal patient selection, IAC remains a vital modality in globe preservation. Its limitations and complications; however, require further optimization, multicenter cooperation, and long-term outcome studies for further optimization of patient safety and survival.

## Figures and Tables

**Table 1:** Demographics, tumor features, and outcome.

Feature		Number	%	Eye Salvage	%	*p* Value
Number	20 Eyes; 67 Injections			11	55%	
Age at diagnosis	median = 19, mean = 21, range 1–72 Months					
Age at the first injection	≤12 months	4	20%	3	75%	0.37
	>12 months	16	80%	8	50%	
Gender	Male	7	35%	3	43%	0.42
	Female	13	65%	8	61%	
Family history	Positive	1	5%	1	100%	0.35
	Negative	19	95%	10	53%	
Laterality	Unilateral	11	55%	6	54%	0.48
	Bilateral	9	45%	5	55%	
Side	Right	11	55%	7	64%	0.40
	Left	9	45%	4	44%	
Indication	Primary	6	35%	5	71%	0.047
	Secondary	14	65%	6	46%	
IIRC stage	C	6	30%	5	83%	0.047
	D	13	70%	6	46%	
	E ^#^	1	5%	0	0%	
TNM stage	cT2a	2	10%	2	100%	0.478
	cT2b	17	85%	9	67%	
	cT3c	1	5%	0	0%	
Subretinal seeds	No	6	30%	4	67%	0.337
	Yes	14	70%	7	50%	
Vitreous seeds	No	12	60%	9	75%	0.046
	Yes	8	40%	3	38%	
Number of injections	≤3 cycles	15	75%	10	67%	0.034
	>3 cycles	5	25%	1	20%	

*p* value of less than 0.05 was considered significant. IIRC: Intraocular Retinoblastoma Classification. ^#^ This patient had the other eye previously enucleated; the remaining eye had neovascular glaucoma.

**Table 2:** Post-treatment outcome and complications (20 eyes, 67 injections).

Outcome (20 Patients)	Number	Percentage
Enucleation	9	45%
High-risk pathological features	0	0%
Orbital recurrence	0	0%
Distant metastasis	1 ^#^	5%
**Systemic Complication (20 Patients)**	**5**	**25%**
Neutropenia	4	20%
Skin erythema	1	5%
**Procedure-related Complications (67 procedures)**	**15**	**22%**
Failure of procedure	5	7%
Ophthalmic arterial spasm	4	6%
Strokes	2	3%
Bronchospasm	4 ^$^	6%
**Ocular Complications (20 Eyes)**	**5**	**25%**
Subretinal hemorrhage	2	10%
Tumor hemorrhage	3 *	15%
Optic atrophy	2	10%
Retinal detachment	2	10%
Severe choroidal and retinal ischemia	2	10%
Peripheral chorioretinal atrophy	3	15%
Oculomotor palsy	1	5%

^#^ This child had the other eye previously enucleated before referral, and no adequate pathology report was available. The other eye received IAC, and the tumor was controlled; however, the patient showed CNS metastasis and passed away. * One had a massive persistent hemorrhage after 1 IAC injection; therefore, the eye was enucleated. ^$^ One procedure was aborted.

## Data Availability

The research data are available upon reasonable request.
